# Supplementary material for: From anatomy to surgical safety: scenario-based learning for internal carotid artery patterns in undergraduate education
Source: BMC Med Educ. 2026 Aug 4;26:1486. doi: 10.1186/s12909-026-10076-2 (PMC13584339; doi:10.1186/s12909-026-10076-2)
Supplement: Supplementary file 1 [file 12909_2026_10076_MOESM1_ESM.pdf]

Article

From Anatomy to Surgical Safety: Scenario-based Learning for Internal Carotid Artery Patterns in Undergraduate Education

Ozden Bedre Duygu [1,*], Figen Govsa [2,*], Tahir Yagdi [3], Ibrahim Duygu [4], Mehmet Asim Ozer [2], and Gokhan Gokmen [5]

[1] Department of Anatomy, Faculty of Medicine, Izmir Bakircay University, 35660 Izmir, Turkey.
[2] Department of Anatomy, Faculty of Medicine, Ege University, 35040 Izmir, Turkey.
[3] Department of Cardiovascular Surgery, Faculty of Medicine, Ege University, Izmir, Turkey.
[4] Department of Cardiology, Faculty of Medicine, Izmir Bakircay University, Izmir, Turkey.
[5] Faculty of Medicine, Dokuz Eylul University, 35220 Izmir, Turkey; ggokmen1999@gmail.com
* Correspondence: ozden2012bedre@gmail.com (O.B.D.); fgovsa@yahoo.com (F.G.); Tel.: +90-5076507873 (O.B.D)

Abstract

Background This study aimed to evaluate the educational value of scenario-based anatomy training using patient-specific three-dimensional models of internal carotid artery (ICA) anatomical patterns.
Methods A total of 120 first-year medical students were assigned to either traditional instruction or scenario-based learning supported by interactive 3D simulators. The training included visualization of ICA configurations such as straight, tortuous, kinking, and coiling patterns using computed tomography angiography data. Students were assessed before and after training using structured questionnaires evaluating anatomical understanding, usability, engagement, clinical relevance, and learning outcomes.
Results The scenario-based group demonstrated statistically significant improvements across all domains ($p < 0.01$–0.001). Anatomical understanding increased from 3.26 ± 0.92 to 4.50 ± 0.78, while clinical relevance improved from 3.12 ± 0.66 to 4.52 ± 0.64. Engagement scores rose from 3.32 ± 0.88 to 4.45 ± ~~0.68~~ 0.70. The overall experience score was higher in the simulation group (4.62 ± 0.64) compared to the traditional group (3.50 ± 0.68).
Conclusion Scenario-based 3D simulation enhances clinical anatomy learning, supports spatial understanding, and improves diagnostic reasoning skills, offering a more effective alternative to traditional teaching methods.
Keywords Internal carotid artery, Computed tomography angiography, Clinical reasoning, Scenario-based training, Simulator, Anatomy training.

Introduction

Anatomy is a foundational discipline for students in healthcare, particularly for those in medical education, as it equips them with essential knowledge of human body structure necessary for understanding clinical conditions [1,2,3]. One of the main goals of anatomical education in modern medical curricula is to provide students with a solid knowledge base that supports the development of clinical reasoning skills and enables them to diagnose and treat diseases effectively. According to a 2023 global survey conducted by the International Federation of Associations of Anatomists, over 90% of medical schools worldwide include anatomy as a core component of their curricula. A solid grasp of anatomical knowledge enables future clinicians to practice safely and effectively, make accurate diagnoses, and perform surgical interventions with competence—critical factors that directly influence patient safety [4].

On the other hand, a topic that has been debated for over 75 years in medical education is knowledge retention. While about two-thirds of unrevised knowledge can be recalled after one year, this rate drops below 50% after two years [4]. Doomernik et al., a decline of approximately 14.7% in anatomical knowledge was observed one and a half years after the initial anatomy course [5]. Ensuring long-term retention is supported by the spacing effect and by clinical-based learning experiences. In a study by case-based anatomical education delivered by surgeons and clinicians, as well as topographical anatomical knowledge, has been shown to significantly enhance long-term retention [6, 7]. Similarly,

the integration of ultrasound laboratories into anatomy education has been found to significantly improve anatomical knowledge in second-year medical students [8]. Although long-term knowledge retention represents an important educational consideration in anatomy training, the present study did not include a longitudinal objective assessment of retention over time. Accordingly, references to knowledge retention in this manuscript are intended to provide a theoretical educational framework based on previous literature rather than to indicate a directly measured outcome of the current study.

Traditional anatomy education relies on lectures, atlases, textbooks, cadaver-based dissections, and digital imaging tools [2, 6, 9, 10]. Although the basic structure of the human body is consistent, individual anatomical variations and patterns are common, and these cannot be fully represented through synthetic models or manikins [11-14]. Each clinical case presents unique anatomical features that demand a thorough understanding of the patient's vascular anatomy [15, 16]. Moreover, every individual possesses distinct anatomical characteristics, including variations in organ structure, congenital anomalies, and developmental malformations, further emphasizing the need for personalized and precise anatomical education [17-19]. Appreciating the hemodynamic and functional consequences of these variations, as well as anticipating potential surgical complications, is essential for successful surgical planning and patient management [20, 21]. Clinical scenario cases delivered through practical applications and facilitated by clinical educators appear to represent a valuable component of the anatomy curriculum.

The present study was designed to evaluate students' perceived educational experiences following scenario-based anatomy training focused on internal carotid artery (ICA) anatomical patterns using patient-specific 3D vascular models derived from CT angiography datasets. Rather than objectively measuring long-term knowledge retention or clinical competency, the study primarily aimed to assess students' perceived anatomical understanding, educational engagement, contextual learning experience, and perceived usefulness of clinically oriented anatomical simulation within undergraduate anatomy education.

Materials and Methods

Study Design and Participants

This study adopts to evaluate the effectiveness of enhanced scenario-based training with a 3D simulator in improving anatomical learning, perception, and student engagement among undergraduate medical students. The training was integrated with four structured clinical case scenarios featuring four anatomical patterns of the cervical part of the internal carotid artery (ICA): straight, tortuous, kinked, and coiled. This study was designed as a comparative quasi-experimental educational intervention. Participants were allocated into two instructional groups according to the predefined educational framework of the course, and no randomization procedure was applied. Pre- and post-intervention comparisons were performed to evaluate educational perceptions and learning-related outcomes between the instructional approaches. To improve methodological transparency, a participant flow diagram illustrating group allocation, participation, and analysis stages was included in accordance with CONSORT-inspired reporting principles adapted for educational research (Figure 1).

Ethical approval was obtained from the Non-interventional Clinical Researches Ethics Committee of Izmir Bakırçay University, and the study was conducted in accordance with the principles of the Declaration of Helsinki (Approval No: 1863–20.11.2024). Participation was voluntary, and informed consent was obtained from all students prior to participation. The study was based on a questionnaire, and no personally identifiable data were collected. CT-A datasets used for three-dimensional model generation were retrospectively obtained from institutional imaging archives following approval by the local ethics committee. All imaging data were fully anonymized prior to segmentation and model development, and no personally identifiable information was accessible to the investigators during the study. The approved protocol permitted the secondary educational use of anonymized imaging datasets for the development of anatomy teaching materials and simulation models.

The study involved 120 first-year medical students (aged 20–23 years, with equal gender distribution). Participants were equally distributed between the traditional anatomy education group (n=60) and the simulator-supported scenario-based learning group (n=60). Participants to whom the anatomical patterns of the cervical ICA region were presented were divided into two groups: one received traditional classical anatomy education, and the other received simulator-supported scenario-based training.

Figure 1: Participant flow diagram of the non-randomized educational intervention study.

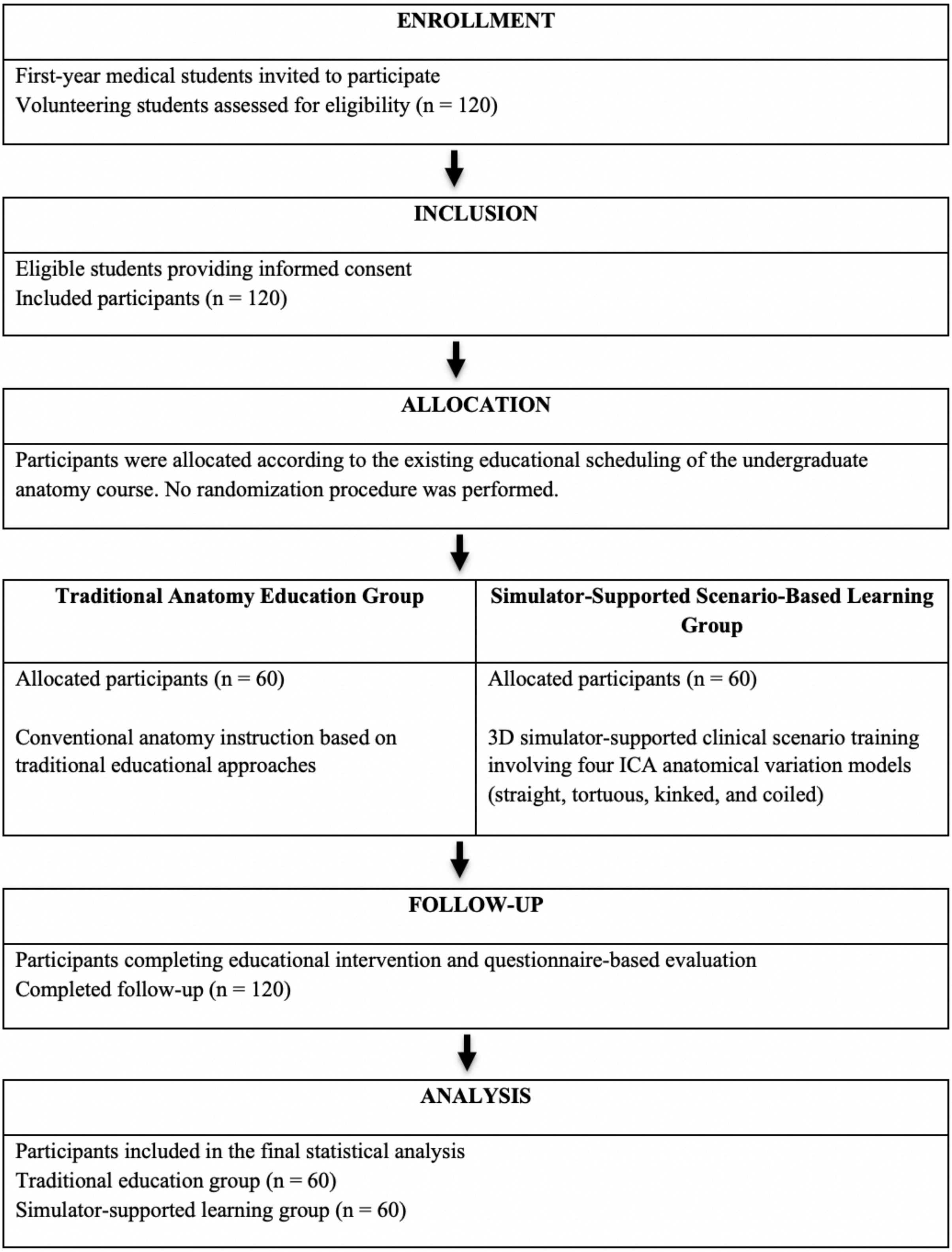

Group allocation was performed according to the existing educational scheduling of the undergraduate anatomy program rather than by randomization. Both groups consisted of first-year medical students who had completed the same core anatomy curriculum prior to participation. The study was designed as a comparative quasi-experimental educational intervention intended to compare students' perceived learning experiences under different instructional approaches. To assess their perceptions of the training, students were asked to complete a survey evaluating their experience with the simulator models in understanding vascular geometry patterns and their clinical relevance in anatomy education (Tables 1, 2). Additionally, focus group interviews were conducted, and the questions students asked experts were documented (Tables 3, 4). Participants were assigned to the educational groups according to scheduled laboratory session availability. No randomization procedure was applied; therefore, the study was designed as a non-randomized quasi-experimental educational intervention.

The study employed a comparative educational intervention framework with non-randomized group allocation based on existing instructional scheduling. Although the intervention included simulator-supported scenario-based activities, the primary objective was a comparative quasi-experimental evaluation of students' perceptions and educational experiences rather than experimental assessment of competency acquisition. Therefore, the study was designed as a non-interventional educational research model. Baseline demographic compatibility between groups was considered during participant distribution to minimize major allocation imbalance.

Table 1. Student Questionnaire on Cervical Region for Internal Carotid Artery (ICA) Learning.

Category		Question	
**Anatomy Understanding**	**1**	To what extent did the CT-A image/3D simulator assist you in identifying whether an anatomical variation of the ICA was present?	Assess the effectiveness of the simulator in detecting the presence of anatomical variations.
	**2**	To what degree did the CT-A image/3D simulator support you in recognizing the exact type of ICA variation?	Evaluate the ability of the simulator to support accurate classification of ICA patterns
	**3**	How effectively did the CT-A image/3D simulator illustrate the differences in ICA anatomical structures?	Measure the simulator's contribution to understanding structural differences in ICA anatomy.
**Usability and Interaction**	**4**	How straightforward was it to investigate the scenario pattern using the CT-A image/3D simulator (e.g., by zooming or rotating)?	Examine clarity of navigation and user-friendliness.
	**5**	Did employing the CT-A image/3D simulator to compare morphological types (normal – tortuous – kinking – coiling) enhance your learning experience?	Assess the added value of interactive features
	**6**	How intuitive did you find the scenario station's interface for exploring anatomical patterns?	Determine the ease of interaction and design efficiency
**Engagement and Interest**	**7**	How stimulating were the scenario-based 3D simulator stations compared	Measure levels of engagement

		with conventional learning tools such as textbooks?	
	**8**	Did the guided use of scenario-based learning encourage you to pursue deeper study of anatomy?	*Evaluate its role in fostering intrinsic motivation*
	**9**	To what extent were you able to maintain interest across the learning sessions while using the 3D simulator stations?	Assess capacity for sustained engagement
**Learning Outcomes**	**10**	How much did your understanding of ICA variations increase following use of the scenario-based station?	Gauge perceived knowledge improvement.
	**11**	To what extent did the scenario improve your ability to retain anatomical knowledge over time?	Assess knowledge retention.
	**12**	Would you consider using this 3D simulator-based scenario again for future anatomy study? Why or why not?	Identify long-term acceptance and preference.
**Educational Value**	**13**	How beneficial were the scenario-based stations for visualizing diverse anatomical conditions such as coiling or kinking?	Assess educational utility for anatomical and clinical variation.
	**14**	Did the 3D simulator-supported scenario aid your comprehension of anatomical relationships in a clinical context, such as surgical planning?	Measure applied clinical relevance.
	**15**	How significant was the 3D simulator-supported scenario in connecting theoretical anatomy to practical clinical applications?	Evaluate its integrative educational value
**Technology and Access**	**16**	How convenient and accessible was the 3D simulator for your learning needs (e.g., device compatibility, remote access)?	Evaluate technical adaptability and ease of access.
	**17**	Did you experience technical issues while engaging with the 3D simulator (e.g., lag, navigation errors)?	Identify challenges affecting the learning process.
	**18**	How dependable was the overall performance of the simulator stations during your study sessions?	Assess technical stability and reliability.
**Personalized Learning Experience**	**19**	How effective was the scenario-based station in adjusting to your individual pace of learning (e.g., adapting quiz difficulty)?	Evaluate personalization in the learning process

	**20**	Did the personalized feedback provided help you concentrate on areas needing improvement?	Assess usefulness of targeted guidance.
	**21**	To what degree did the adaptive features of the station enrich your learning experience overall?	Measure perceived benefit of adaptive personalization.
**Clinical Reasoning and Application**	**22**	How useful were the clinical case studies in enabling you to apply anatomical knowledge to real-life situations (e.g., ICA coiling, kinking)?	Evaluate contribution to clinical reasoning.
	**23**	Did the scenario-based station improve your ability to design or understand preoperative planning using anatomical models?	Assess its impact on surgical preparation.
	**24**	After engaging with the clinical case studies, how confident do you feel in interpreting various ICA patterns (straight, tortuous, kinking, coiling)?	Measure diagnostic readiness
**Collaboration and Peer Feedback**	**25**	How effective was the peer feedback provided through the station in developing your presentation skills?	Assess influence on communication abilities.
	**26**	Did the station's collaborative tools (e.g., peer discussions, focused feedback, expert interactions) enhance your interdisciplinary communication?	Evaluate collaborative learning outcomes
	**27**	How valuable was the automated feedback on your peer interactions for supporting your learning process?	Measure contribution of automated peer-feedback systems.
**Overall Experience**	**28**	On a scale of 0 to 5, how would you evaluate your overall learning experience with the scenario-based 3D simulator for anatomy?	Assess the overall effectiveness and quality of the user experience
	**29**	Would you support the inclusion of this station as a regular component of the anatomy curriculum?	Examine satisfaction levels and the potential for widespread implementation in medical education
	**30**	What further developments or additional functions would you recommend to increase the effectiveness of the simulator-based station?	Collect suggestions for improving the station and its educational modules.

Table 2. Pre- and Post-Training Comparison of Anatomical Knowledge Regarding Internal Carotid Artery Patterns.

Category	Learning Group	Pre-Activity Mean (±SD)	Post-Activity Mean (±SD)	Statistical Significance (p-value)
**Anatomy Understanding**	Scenario-based	3.26 ± 0.92	4.50 ± 0.78	p < 0.001
	Traditional	3.05 ± 0.78	3.28 ± 0.72	p = 0.10
**Usability and Interaction**	Scenario-based	3.58 ± 0.87	4.70 ± 0.68	p < 0.01
	Traditional	3.42 ± 0.73	3.58 ± 0.67	p = 0.29
**Engagement and Interest**	Scenario-based	3.32 ± 0.88	4.45 ± 0.70	p < 0.001
	Traditional	3.05 ± 0.73	3.40 ± 0.78	p = 0.15
**Learning Outcomes**	Scenario-based	3.12 ± 0.68	4.66 ± 0.72	p < 0.001
	Traditional	3.18 ± 0.74	3.45 ± 0.68	p = 0.08
**Educational Value**	Scenario-based	3.28 ± 0.92	4.60 ± 0.66	p < 0.01
	Traditional	3.15 ± 0.67	3.27 ± 0.64	p = 0.12
**Technology and Access**	Scenario-based	3.48 ± 0.83	4.55 ± 0.43	p < 0.001
	Traditional	3.25 ± 0.68	3.60 ± 0.59	p = 0.19
**Clinical Relevance**	Scenario-based	3.12 ± 0.66	4.52 ± 0.64	p < 0.001
	Traditional	3.05 ± 0.71	3.23 ± 0.63	p = 0.13
**Personalized Learning Experience**	Scenario-based	3.35 ± 0.81	4.50 ± 0.65	p < 0.001
	Traditional	3.21 ± 0.76	3.31 ± 0.70	p = 0.25
**Collaboration and Peer Feedback**	Scenario-based	3.32 ± 0.79	4.58 ± 0.64	p < 0.001
	Traditional	3.20 ± 0.76	3.42 ± 0.69	p = 0.15
**Overall Experience**	Scenario-based	3.38 ± 0.85	4.62 ± 0.64	p < 0.001
	Traditional	3.24 ± 0.82	3.50 ± 0.68	p = 0.17

Notes: The scenario-based learning group shows significant improvements (p<0.001) across all domains, particularly in learning outcomes (4.66 ± ~~0.62~~ 0.72) and clinical relevance (4.52 ± 0.64), while the traditional learning group's gains are minimal and non-significant.

Selection of the Sample Cases

Radiological Evaluation

ICA cases were selected from the CT-A archive of the Department of Cardiovascular Surgery at Ege University Hospital (Fig. 2). As this study is not a clinical trial, clinical trial registration is not applicable. The cases involved patients between 18 and 62 years of age. The anatomical geometric patterns of the cervical segment of the ICA were classified into four categories: straight (no angulation) (Figs. 3A,B), tortuosity (15–60 degrees)(Figs. 4A,B), kinking (90–145 degrees) (Figs. 5A,B), and coiling (360 degrees) (Fig. 6B). The coiling type of ICA was characterized by a distinct 360-degree loop [8,9]. Representative cases corresponding to each of these geometric patterns were included in the study.

Image Post-Processing and Segmentation

A 64-slice CT-A scan was used to describe the anatomical characteristics of the ICA patterns (Figs. 3-5). A threshold of approximately 150–400 Hounsfield units was applied to remove soft tissue and bone. Three-dimensional anatomical models were generated from CT-A datasets using a multi-step digital workflow involving DICOM segmentation, STL conversion, mesh optimization, and additive manufacturing preparation. Image processing and segmentation were performed using Analyze 12.0 and 3D Slicer software, whereas mesh refinement and print optimization were conducted using Autodesk Meshmixer and Simplify3D platforms.

1. The original CT-A images, in DICOM (Digital Imaging and Communications in Medicine) format, were transferred to a dedicated workstation and processed using Analyze 12.0 software.
2. The DICOM files were converted to stereolithography (STL) format using 3D Slicer (version 4.10.1), an open-source station for medical image computing.
3. The STL files were refined and optimized using Autodesk Meshmixer (version 3.4.35).
4. The finalized models were prepared for 3D printing using Simplify3D software (version 4.0).

Creating ICA Pattern Simulator Models

The straight, tortuous, kinked, and coiled ICA patterns were printed using a Mass Portal Pharaoh XD 20 3D printer to produce accurate, life-sized anatomical models (Fig. 6). The selected ICA anatomical configurations (straight, tortuous, kinking, and coiling) were chosen because they represent the most clinically recognized variations of the cervical internal carotid artery and encompass a broad spectrum of geometric, hemodynamic, radiological, and surgical characteristics. Their inclusion allowed students to compare normal anatomy with progressively more complex vascular patterns relevant to clinical practice, diagnostic interpretation, and surgical decision-making.

Development of the Scenarios

Four case scenarios were developed collaboratively by experts in anatomy, cardiology, cardiovascular surgery, and medical modeling. Each scenario presented a distinct ICA anatomical pattern along with its corresponding clinical anatomy. For each scenario, participants had access to textual information, CT-A imaging, and a 1:1 scale solid anatomical simulator for hands-on examination (Fig. 7). Both groups were exposed to the same four cervical ICA anatomical patterns (straight, tortuous, kinked, and coiled) during training sessions. Educational sessions were conducted under comparable classroom conditions and within equivalent time intervals.

An assessment station was used to compare the educational value and diagnostic contribution of each imaging modality across various parameters. The traditional education group received conventional anatomy instruction based on standard theoretical lectures and CT-A image interpretation, whereas the scenario-based group additionally interacted with patient-specific 3D printed models and guided case-based discussions. Experts in each session encouraged students to ask questions related to the scenarios. The scenario-based intervention consisted of multiple integrated educational components, including interdisciplinary expert interaction, active learning activities, peer discussion, CT-A image interpretation, hands-on examination of tactile 3D vascular models, and clinically oriented reasoning exercises. Therefore, the observed educational outcomes should be interpreted as the combined effect of a multifaceted instructional framework rather than the isolated contribution of the 3D simulator alone.

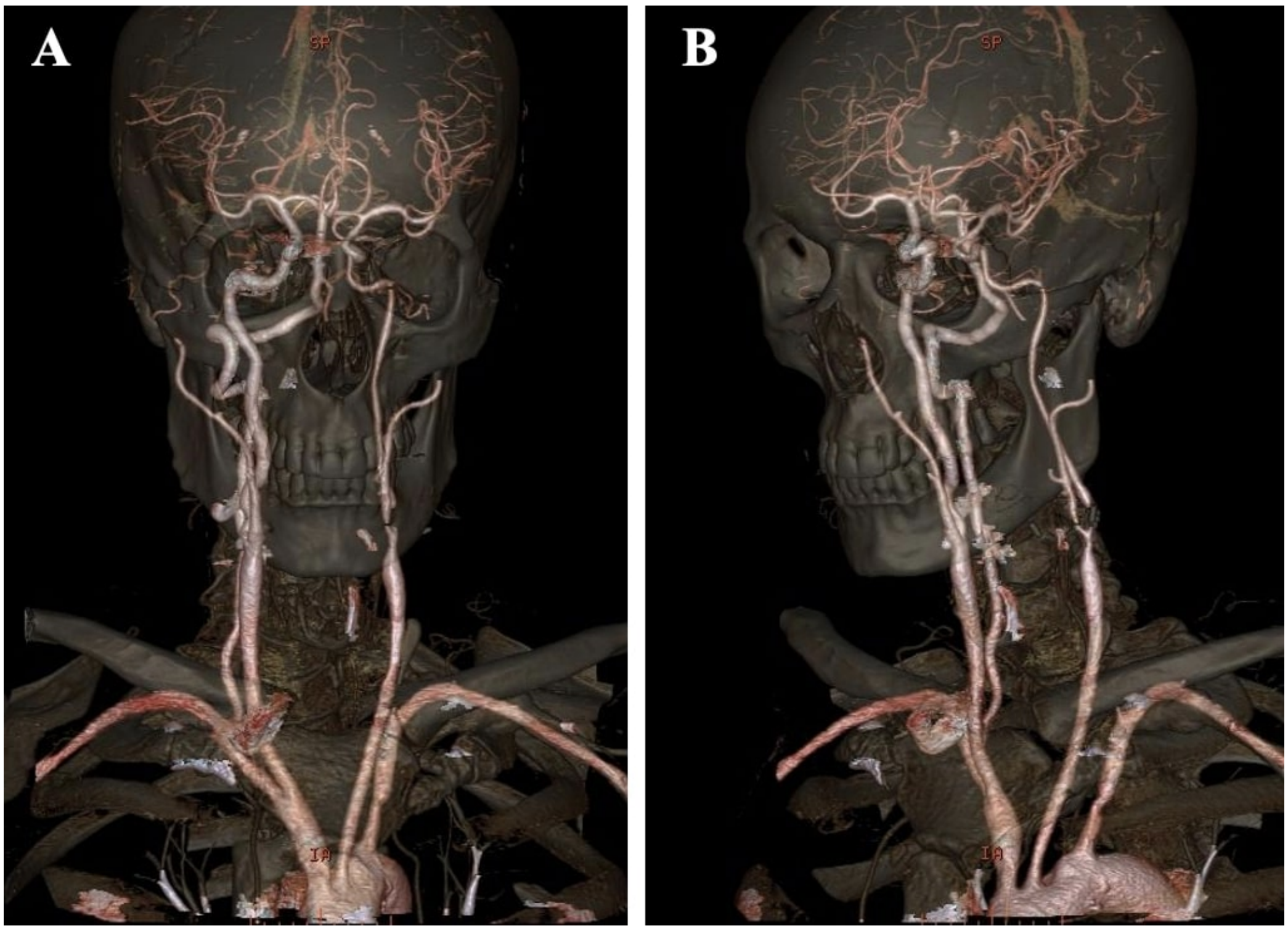

Figure 2. Original CT angiography images of the head and neck region. **A.** Frontal view, **B.** Sagittal view.

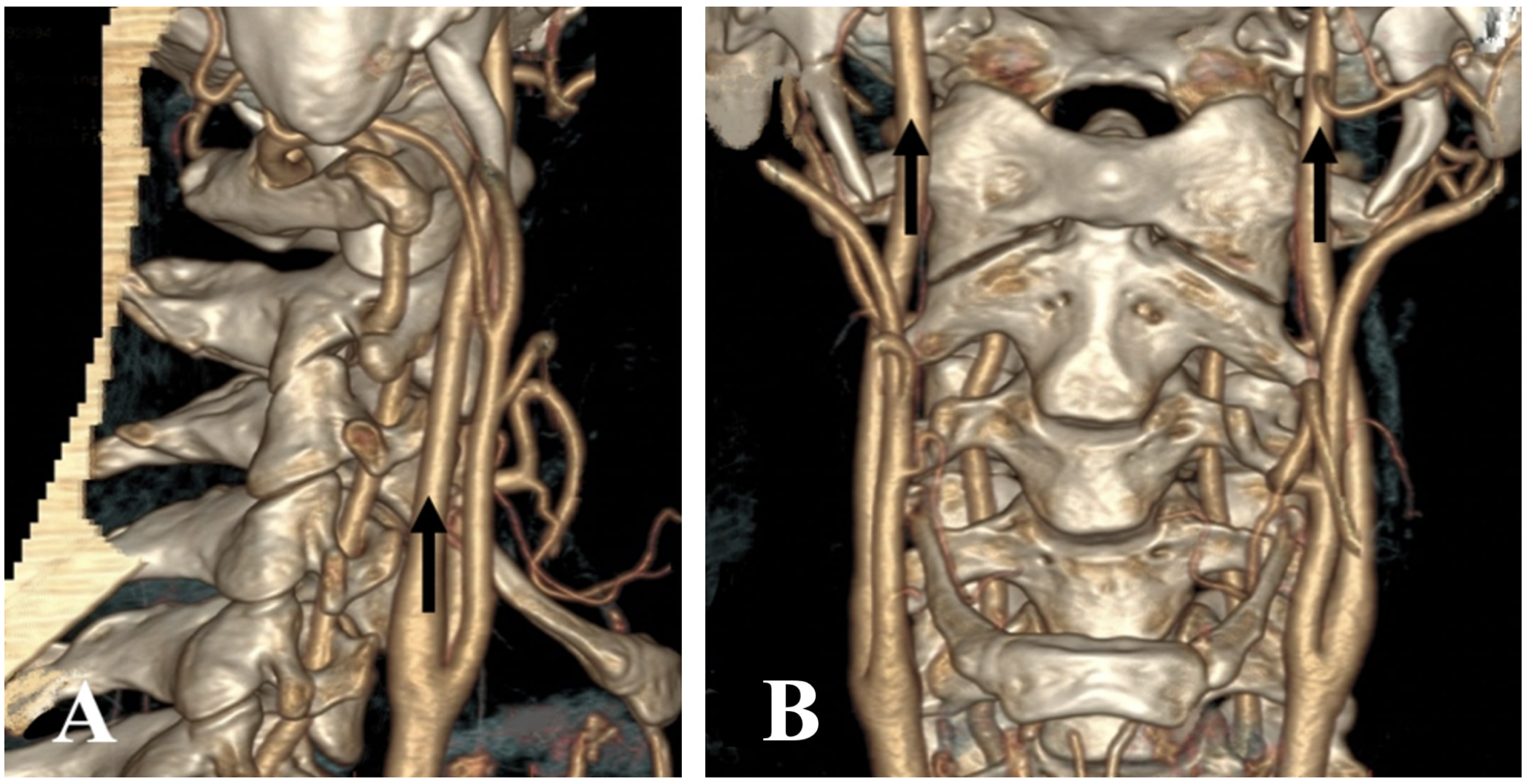

Figure 3. CT angiography showing the **straight pattern** (**black arrow**) of the cervical portion of the internal carotid artery. **A.** Sagittal view, **B.** Frontal view.

Survey

The educational intervention consisted of a scenario station designed to facilitate anatomy learning, perception, and engagement. ~~The same perception survey questions were administered to both the traditional and scenario-supported student groups, both before and after the training.~~ The same core perception questionnaire was administered to both the traditional and scenario-based learning groups before and after the educational intervention. The same core questionnaire was administered to both the traditional and scenario-based learning groups. The questionnaire structure and educational domains were identical for both groups; however, several items were contextually adapted to reflect the specific instructional characteristics of the scenario-based intervention. Comparative statistical analyses were

performed only on the shared educational domains that were applicable to both groups. No previously validated questionnaire was available for this specific educational setting; therefore, a study-specific instrument was developed and subjected to expert review and internal consistency analysis. Questionnaire items were designed to evaluate perceived educational experience and clinically contextualized anatomical understanding rather than objective surgical competency or professional performance. Participants completed a multi-item survey focusing on understanding vascular pattern anatomy, recognizing ICA variations, classifying patterns, making independent decisions, performing differential pattern diagnosis, planning key clinical steps, anticipating unexpected events, and understanding the need for endovascular surgery — all aimed at assessing their perceptions of anatomical training (Tables 1 and 2). Each educational session lasted approximately 30 minutes per anatomical station. Both educational groups completed the sessions within the same instructional period and under comparable educational conditions. Because the study primarily relied on self-reported questionnaire responses and focus group evaluations, no formal evaluator blinding procedure was applied. However, all participants completed the survey individually under standardized instructional conditions.

The questionnaire was structured to evaluate multiple educational domains, including anatomical understanding, usability, engagement, learning outcomes, clinical relevance, interdisciplinary interaction, and overall educational experience. The survey items were developed according to the educational objectives of the scenario-based station and reviewed collaboratively by experts in anatomy, cardiovascular surgery, and medical education to ensure content appropriateness and conceptual consistency. The 30-item structured questionnaire used in this study was newly developed specifically for the educational objectives of the present investigation. Because no previously validated questionnaire was available for this specific educational context, a study-specific instrument was developed and subsequently subjected to expert review and internal consistency analysis. Questionnaire content was prepared according to anatomy education principles, scenario-based learning objectives, and expert academic feedback. Content validity was established through expert review, and internal consistency analysis demonstrated acceptable Cronbach's alpha values for the questionnaire domains, supporting the reliability of the instrument within the context of this educational intervention. The questionnaire demonstrated good internal consistency across all domains, with Cronbach's alpha coefficients of 0.84 for Anatomical Understanding, 0.82 for Usability and Interaction, 0.87 for Learning Outcomes, 0.85 for Clinical Relevance, and 0.86 for the overall instrument. Because the questionnaire primarily aimed to assess perceived educational benefit within a pilot educational framework, the findings should be interpreted as perception-based outcomes rather than objective measurements of clinical competency or diagnostic performance.

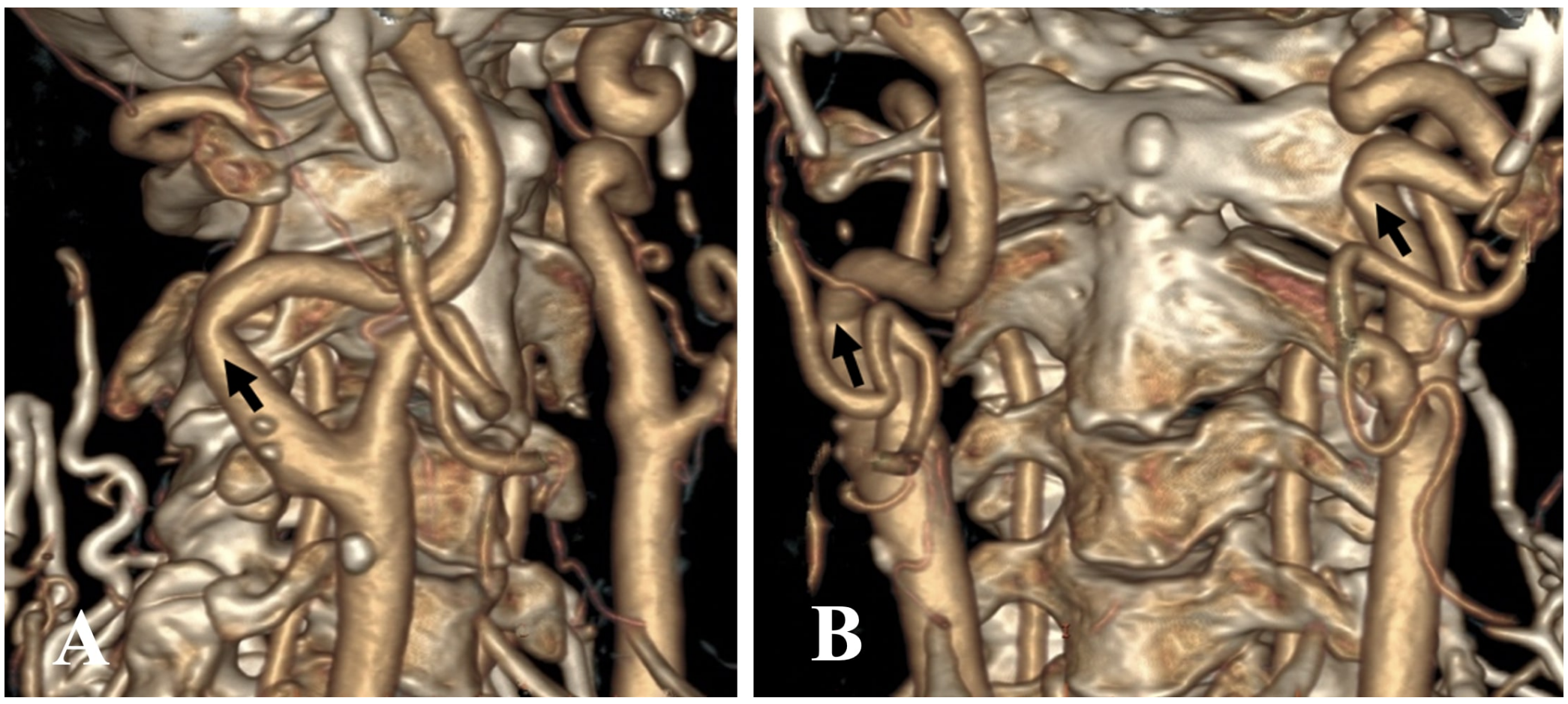

Figure 4. CT angiography showing the **tortuous pattern** (**black arrow**) of the cervical portion of the internal carotid artery. **A.** Sagittal view, **B.** Frontal view.

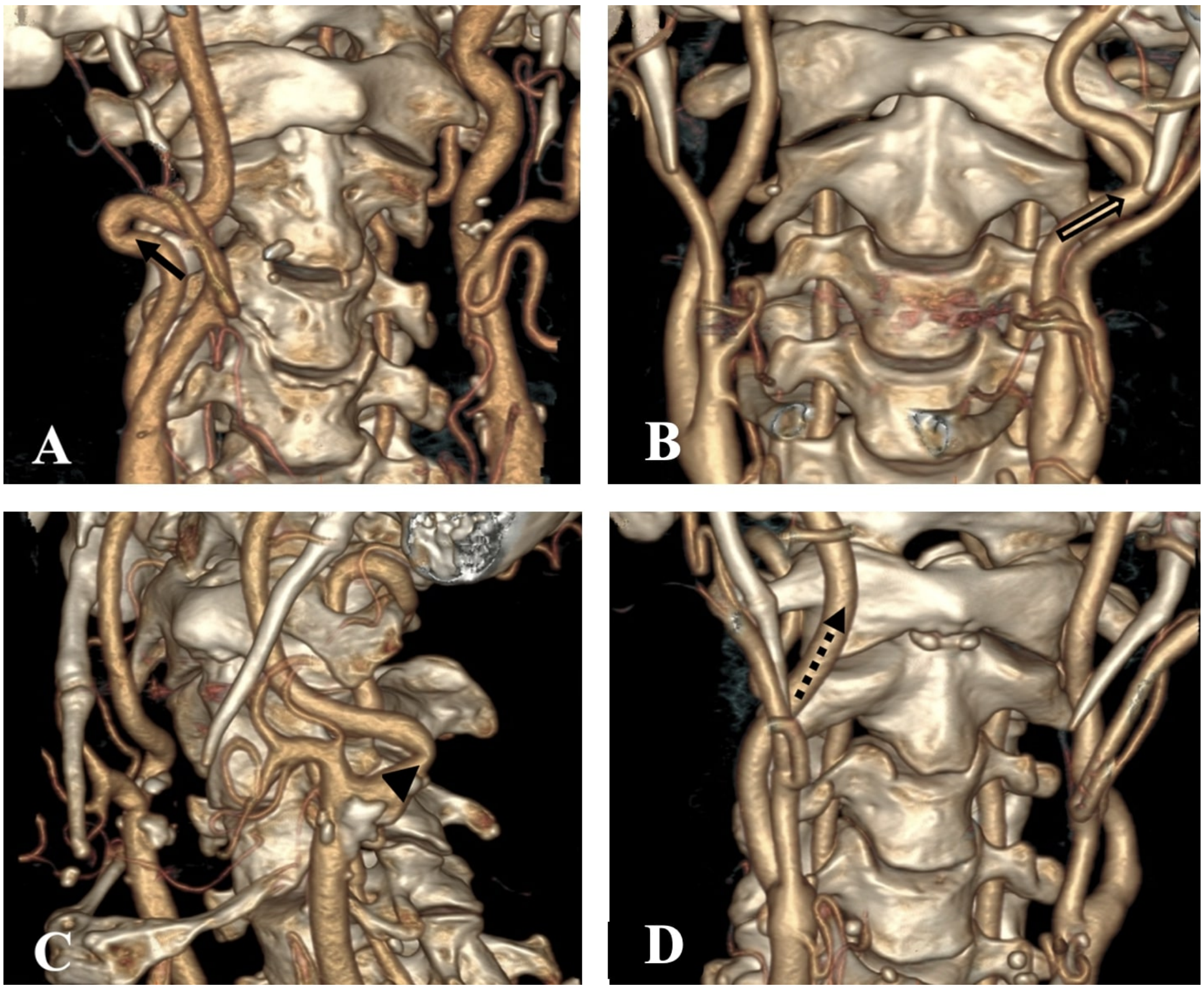

Figure 5. CT angiography of the cervical portion of the internal carotid artery.
A and B: Kinking pattern (black arrow), **C and D:** Coiling pattern (black arrow),
A and C: Sagittal views, **B and D:** Frontal views.

In addition to the survey, open-ended focus group interviews with students and experts were conducted, and the questions posed by students to the experts during the scenario-based training sessions, along with the answers, were recorded (Tables 3 and 4). The study team collected data in three phases from the participants. Students were given 30 minutes for each station, starting from the first, and were asked to rate both the CT-A imaging and the 3D simulator on a 5-point scale, where 0 represented the lowest and 5 the highest score. Survey results were evaluated for each parameter and participant individually. The study procedure consisted of participant allocation, educational exposure, questionnaire administration, focus group interviews, and comparative evaluation of the obtained data between groups. Participation in the study was voluntary. First-year medical students who agreed to participate and met the inclusion criteria were enrolled in the study. Participants were allocated to the traditional or simulator-supported educational groups according to the existing educational scheduling of the undergraduate anatomy course. No randomization procedure was performed. A participant flow diagram illustrating enrollment, allocation, follow-up, and final analysis has been provided (Figure 1).

Descriptive statistics and the Wilcoxon signed-rank test were used for within-group comparisons between pre- and post-intervention questionnaire scores (IBM Corp., Armonk, NY, USA). Because questionnaire responses were collected using a five-point ordinal Likert scale from the same participants before and after the educational intervention, a non-parametric paired analysis was considered appropriate. Statistical significance was set at $p < 0.05$. Statistical analyses focused on within-group and between-group comparisons of questionnaire-based educational outcomes. Given the exploratory educational nature of the study and the perception-based assessment design, effect size calculations

and psychometric validation analyses were not included. The results therefore reflect comparative educational tendencies rather than definitive causal inferences regarding clinical reasoning or procedural competency development.

Results

The study employed a non-randomized comparative quasi-experimental educational design conducted at a single center.

Creating Life-Size, Patient-Specific 3D simulator

The 3D simulator provided an outstanding display of the anatomy of ICA variation patterns, offering clear and comprehensive views of the related scenario cases (Figs. 3-6). The production time for each model was approximately 2.5 hours for design and 72 hours for printing, with a printing cost of around 50 euros.

Measurement

Structural measurements obtained from the CT scans of the simulator models and the original CT-A images were statistically compared. The absence of any significant difference ($p > 0.05$) demonstrated that the models were true 1:1 representations specific to ICA coiling pattern cases.

Scenarios

ICA Straight (Normal) Pattern Clinical Anatomy Scenario

Case 1: A 45-year-old woman presenting with persistent headache underwent head and neck imaging at the neurology outpatient clinic. The ICA was found to have a straight (normal) pattern. She had no other medical history, did not smoke, exercised regularly, and maintained a healthy diet. Systemic examination was normal, and no carotid bruit was detected. CT-A showed that both right and left ICAs followed a straight course with a slight curvature from the carotid bifurcation to the carotid canal (straight pattern) (Fig. 2,3,6A). The lumen diameter and carotid bifurcation were normal, with no plaques or stenosis. The 3D simulator demonstrated a straight cervical segment of the ICA (Fig. 6A) with laminar flow and no evidence of turbulence.

Clinical anatomical evaluation: The anatomy expert (O.B.D.) guided students to focus on the straight ICA anatomy in this scenario. They studied how this anatomical pattern preserves laminar flow and provides a balanced anatomical relationship with surrounding tissues.

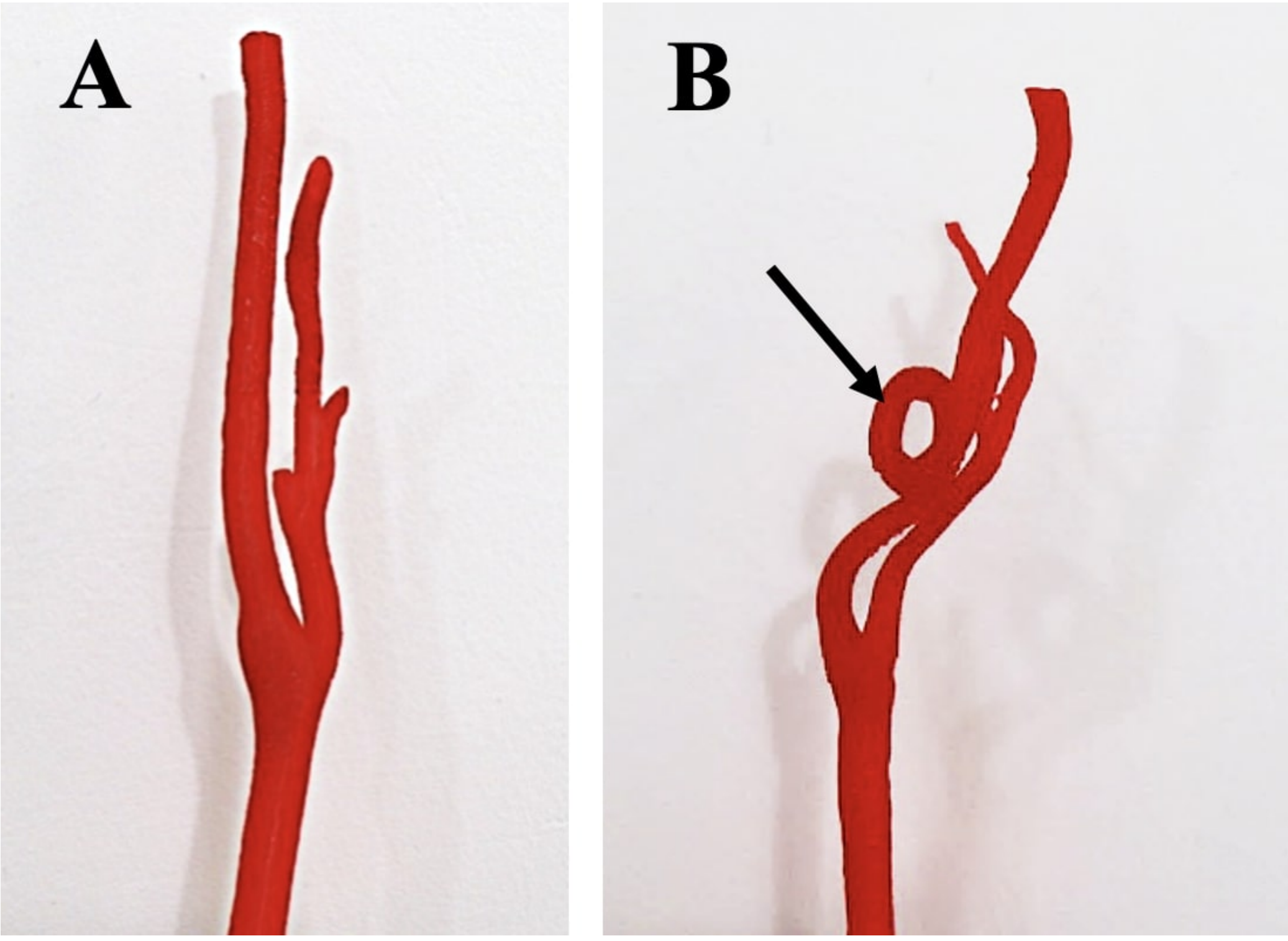

Figure 6. Scenario-based simulator views of the cervical portion of the internal carotid artery. **A:** Straight pattern, **B:** Coiling pattern.

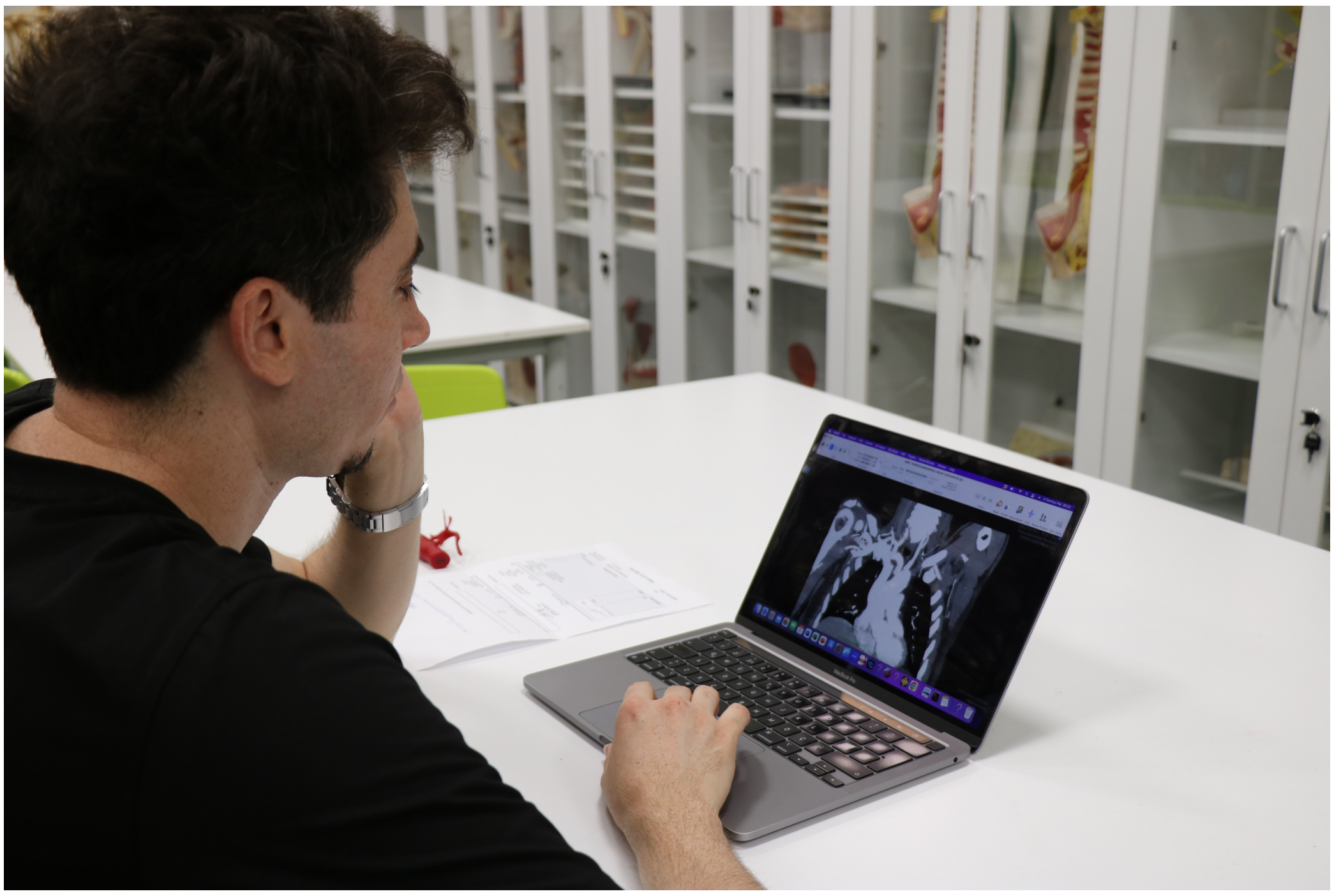

Figure 7. Cardiologist reviewing CT angiography within the scenario-based simulator station.

ICA Tortuosity (15–60°) Pattern Clinical Anatomy Scenario

Case 2: A 68-year-old man reported three months of dizziness and brief episodes of blurred vision, particularly when turning his head side to side. He sometimes experienced a sense of ear fullness during dizziness. His medical history included hypertension and type 2 diabetes. Physical and systemic examinations were normal, and no carotid bruit was detected. CT-A revealed approximately 35° tortuosity of the right ICA at the distal cervical segment (C2–C3 level), without significant lumen narrowing (<30%) or atherosclerotic plaque (Fig. 4A,B). The 3D simulator displayed a mild

S-shaped or C-shaped curvature of the right cervical ICA.

Clinical anatomical evaluation: The anatomy expert (F.G.) demonstrated the changes in ICA geometry using CT-A and the simulator, emphasizing how head rotation and extension might bring the tortuous segment closer to surrounding tissues, contributing to symptoms. It was explained that such anatomical variations could cause transient ischemic symptoms but did not indicate a need for endovascular surgery. Patients were advised to avoid excessive head movements and control risk factors. Using a 3D simulator, it was demonstrated how laminar flow could be disrupted and turbulence could develop in the tortuous cervical ICA segment.

ICA Kinking (90–145°) Pattern Clinical Anatomy Scenario

Case 3: A 72-year-old woman presented with sudden-onset, transient unilateral weakness and speech difficulty. That morning, she had experienced weakness in her left arm and slurred speech lasting about five minutes. Her history included hypertension, hyperlipidemia, and a 20-year smoking history. Family history was positive for ischemic stroke. No carotid bruit was heard. CT-A showed approximately 110° kinking of the right ICA at the cervical segment (C3 level), with a posterolateral bend (Fig. 5A,B). Flow jet phenomena causing transient narrowing and mild post-stenotic dilation were observed at the kink, with no significant plaque at the carotid bifurcation (Fig. 5A,B). The 3D simulator displayed a sharply angulated segment of the right cervical ICA, illustrating a kink rather than a curve. The proximal and distal segments had a wide arterial profile.

Clinical anatomical evaluation: The cardiology expert (I.D.) explained how the kinked segment's sensitivity to head movements could relate to the patient's symptoms (Fig. 7). The kinking could cause compression of adjacent soft tissues, including potential entrapment between the internal jugular vein, vagus nerve, and sternocleidomastoid muscle. Surgery was not indicated; patients were advised to avoid excessive head movements and manage risk factors. Using a 3D simulator, it was demonstrated how laminar flow could be disrupted and turbulence could develop in the kinking cervical ICA segment.

Table 3. Selected Student Quotes Categorized by Thematic Analysis and Expert Focus Group Findings.

**Item**	Opportunities for hands-on practice
**1**	"Before the scenario, I was really struggling because you are expected to identify whether a pattern is ICA kinking or coiling just by looking at a flat, single-plane image. But when I held the simulator and rotated it, I could better understand the geometric change in the vessel. It's not the same at all."
**2**	"The scenario-based training was very valuable. At the very least, focusing on the CT-A images was really helpful because we'll probably need to do that in the future."
**3**	**"After practicing with the scenario, I felt much more prepared to identify ICA patterns. Honestly, before the scenario-supported training, it wasn't easy for me to relate a 2D image to a 3D structure. With the simulator support, I felt confident I could manage it if asked in an exam."**
	**Reinforcement of knowledge and contextualization**
**4**	"The ICA vessel pattern types only truly make sense when you bring them all together. At first, I realized how little I knew and how hard it was, but after the scenario-based training, I felt much more prepared for the exam."
**5**	"I started to take more interest in the clinical anatomy of the patterns, so going back over the material became easier and more motivating."

**6**	**"I think the value of these scenarios is that they give context to what the anatomy knowledge is actually for. It trains your brain to recall the right information at the right time—just like being in a real situation. I think it's very beneficial for long-term memory."**
	**Diverse perspectives and educator variety**
**7**	"Listening to the surgeon talk about surgical aspects was really impressive. I really appreciated the opportunity to learn from and interact with a surgeon."
**8**	**"Each station was quite different from the previous one, and I thought that was fantastic. The expert running each station was very good at encouraging active participation. It was truly exceptional and very different from a traditional anatomy lab."**
**9**	"Having clinicians from different specialties there was very valuable and impactful. They highlighted what to look for and pointed out all the clinical connections."
**10**	"Learning about the ICA anatomical patterns from both a cardiologist and a cardiovascular surgeon was so important."
	**Application of anatomy in a clinical context**
**11**	**"Everything we learned had a clinical meaning and place, and we learned how that knowledge could be applied in a clinical setting. That was very important."**
**12**	"There was a great bridge between ICA anatomy and its endovascular surgery applications. It was really good to see these concepts applied in practice."

ICA Coiling (360°) Pattern Clinical Anatomy Scenario

Case 4: A 65-year-old man presented with dizziness and imbalance triggered by sudden head movements. He reported brief dizziness and lightheadedness when rapidly turning or extending his head and described an episode of sudden dizziness and falling that day. He had hypertension and mild carotid atherosclerosis. Systemic examination was normal, and no carotid bruit was heard. CT-A revealed a 360° complete spiral (coiling) of the right ICA at the cervical segment (C2 level) (Fig. 5C,D). Flow velocity was reduced at the coiling site, with minimal compensatory dilation distal to the coiling. Lumen narrowing was not significant (<30%), and no meaningful plaque was noted at the bifurcation. The 3D simulator clearly demonstrated the cervical ICA's 360° spiral coiling pattern (Fig. 6B). The coiling segment was positioned such that head flexion and extension could bring it closer to surrounding structures (especially prevertebral muscles and the lateral pharyngeal wall), potentially causing transient flow changes and cerebral hypoperfusion.

Clinical anatomical evaluation: The cardiovascular surgery expert (T.Y.) emphasized the O-shaped configuration of the cervical ICA in this pattern. The coiling segment's sensitivity to head extension and rotation was explained, highlighting the risk of transient mechanical compression. Surgery was not indicated; patients were advised to avoid excessive head movements and control vascular risk factors. Using the 3D simulator, it was demonstrated how laminar flow could be disrupted and turbulence could develop in the coiling segment of the cervical ICA.

Pre- and Post-Intervention Learning Outcomes

The responses to the scenario survey questions are presented in Table 1 and 2. The majority of students who participated in the scenario-based training on ICA patterns evaluated the station as providing an excellent learning experience for understanding ICA anatomy.

The group that received scenario-based training demonstrated significant improvements across all evaluated domains ($p < 0.01$ or $p < 0.001$). Notably, their anatomical understanding increased from 3.26 ± 0.92 to 4.50 ± 0.78, clinical relevance rose from 3.12 ± 0.66 to 4.52 ± 0.64, and collaboration and peer feedback scores improved from 3.32 ± 0.79 to 4.58 ± 0.64. These results suggest a positive educational influence on students' perceived preparedness and contextual understanding within anatomy education. Engagement and interest also showed a marked increase, from 3.32 ± 0.88 to

4.45 ± 0.70 ($p < 0.001$), reflecting high levels of student motivation. Furthermore, usability and interaction (4.70 ± 0.68) and technology and access (4.55 ± 0.43) ratings underscored the station's accessibility and user-friendliness. By contrast, the traditional group, which received traditional instruction, showed only slight and statistically non-significant gains in all areas (e.g., anatomical understanding: 3.05 ± 0.78 to 3.28 ± 0.72, $p = 0.10$; clinical relevance: 3.05 ± 0.71 to 3.23 ± 0.63, $p = 0.13$). The scenario-based group's overall experience score (4.62 ± 0.64; $p < 0.001$) was substantially higher than that of the traditional group (~~3.40~~ 3.50 ± 0.68; $p = 0.17$), further confirming the station's superior educational value in enhancing anatomical knowledge, clinical reasoning, and teamwork skills within medical training (Table 2).

Table 4. Expert Feedback on Student Questions and Their Interaction with the Scenario.

**Question/Answer**	
Question 1:	What is the risk of a normal (straight) internal carotid artery (ICA) being affected by head and neck movements?
**Answer 1**	A straight ICA is not significantly affected by head and neck movements. Its linear course minimizes the risk of transient flow disturbances or mechanical compression during head position changes. Laminar flow is maintained in this pattern, with no turbulence or embolic risk, and the vessel maintains a balanced anatomical relationship with surrounding structures.
Question 2:	In a normal ICA, where is the risk of atherosclerotic plaque formation highest?
**Answer 2**	In a normal ICA, the carotid bifurcation region (carotid sinus) is most susceptible to plaque development due to turbulence. The straight segment, where laminar flow predominates, has a lower risk of plaque formation.
Question 3:	What is the surgical significance of a straight ICA?
**Answer 3**	A straight ICA is typically found in its expected anatomical location during pharyngeal surgeries and neck dissections. Unlike other patterns, it does not extend into the retropharyngeal space, reducing the risk of vascular injury during such procedures.
Question 4:	What neurological symptoms can be associated with ICA tortuosity?
**Answer 4**	ICA tortuosity may present with symptoms of transient ischemic attacks, including dizziness, transient vision loss (amaurosis fugax), temporary hemiparesis, and speech disturbances.
Question 5:	Which anatomical structures can interact with a tortuous ICA?
**Answer 5**	A tortuous ICA segment may come into close proximity with the internal jugular vein, vagus nerve, sternocleidomastoid muscle, and lateral pharyngeal wall, increasing the risk of transient vessel compression during head movements.
Question 6:	Where is ICA kinking most commonly observed?
**Answer 6**	ICA kinking most often occurs in the cervical segment, particularly at the C2–C3 vertebral level and proximal to the carotid bifurcation.
Question 7:	What is the clinical consequence of ICA kinking during head and neck movements?
**Answer 7**	Head rotation or extension can cause transient narrowing at the kinked segment, potentially leading to cerebral hypoperfusion, transient ischemic attack symptoms, and, rarely, syncope.

Question 8:	What is the significance of ICA kinking in terms of thrombosis and embolism?
**Answer 8**	The kinked segment can generate turbulent flow and low shear stress, creating a predisposition for thrombus formation and microembolization.
Question 9:	How can kinking be distinguished from tortuosity?
**Answer 9**	Tortuosity typically presents as smooth S- or C-shaped curves with angulation between 15° and 60°. In contrast, kinking forms a sharp angle between 90° and 145°, giving the appearance of a “bent” or “broken” vessel.
Question 10:	What is the surgical relevance of a coiled ICA?
**Answer 10**	Coiling may position the ICA in an unexpected location, often closer to the retropharyngeal space. This increases the risk of vascular injury during pharyngeal surgeries (e.g., tonsillectomy, abscess drainage), anterior cervical procedures, and neck dissections.

Qualitative Feedback and User Perceptions

General observation: The scenario-based training group showed significant improvement across all learning domains. The p-values ($p < 0.001$ or $p < 0.01$) indicate that the differences observed in most categories were statistically highly s ignificant (Table 2). The domains with meaningful improvement included anatomical understanding, usability and interaction, engagement and interest, learning outcomes, educational value, technology and access, clinical reasoning, personalized learning experience, collaboration and peer feedback, and overall experience. A p-value below 0.05 suggests that these results are unlikely to have occurred by chance and reflect the positive impact of the 3D simulator-supported scenario-based learning intervention.

In contrast, the control group receiving traditional training showed only minor, statistically non-significant improvements across all categories, with p-values generally above 0.05. These results indicate no meaningful development in the control group.

Educational conclusion: The data clearly demonstrate that simulator-supported scenario-based anatomy training led to significant improvements in all learning domains, while no substantial progress was observed in the control group. This highlights the effectiveness of the intervention. The findings indicate improved student perceptions regarding clinically contextualized anatomical understanding and perceived clinical reasoning processes within the educational setting.

Thematic Analysis and Expert Focus Group Findings

The thematic analysis of expert focus group interviews, summarized in Table 3, reflects key themes identified from feedback provided by the target group at the conclusion of the intervention. The main themes that emerged included:

- Opportunities for hands-on practice,
- Reinforcement of anatomical knowledge and contextualization through clinical reasoning,
- Interaction with educators from different clinical specialties, such as anatomy, cardiology, and cardiovascular surgery,
- The ability to apply anatomical knowledge in a clinical context.

An analysis of Table 3 demonstrates that it holds value both as an educational tool and as a framework for clinical risk assessment. The findings indicate that students reported improved ability to relate anatomical knowledge to clinically contextualized scenarios.

Expert Feedback on Student Questions and Scenario Interaction

Experts involved in the scenarios reported that students asked more advanced and clinically meaningful questions compared to a typical anatomy laboratory setting. Expert comments and scenario station notes provided detailed insights into these high-level clinical anatomy questions (Table 4).

An analysis of Table 4 demonstrates that it holds value both as an educational tool and as a framework for clinical risk assessment. The findings indicate that students are able to integrate anatomical knowledge into a clinical context, while expert feedback serves to further enhance and deepen the learning process.

Table 4 classifies anatomical variations within the framework of normal – tortuous – kinking – coiling. This provides a systematic structure from an educational perspective.

- Straight ICA (Q1–Q3): Risk factors (e.g., motion, plaque formation), surgical safety, and anatomical location
- Tortuous ICA (Q4–Q5): Neurological symptoms (e.g., TIA findings), interaction with neighboring structures
- Kinking (Q6–Q9): Localization, hemodynamic effects (e.g., hypoperfusion, embolism risk), morphological differentiation criteria (kinking vs tortuosity)
- Coiling (spiraled ICA) (Q10): Surgical risks (e.g., retropharyngeal positioning → risk during tonsillectomy, drainage, etc.)

In the pedagogical analysis of scenario-based learning based on student questions and learning outcomes in Table 4:

- Level of student questions: The questions are not limited to "what" inquiries, but also stimulate higher-order thinking such as clinical risks, surgical significance, and distinguishing features. This indicates that students are focused not only on anatomy but also on clinical correlations.
- Quality of expert responses: The responses are clear, precise, and based on technical terminology. Each variation is addressed in terms of its hemodynamic (laminar/turbulent), clinical (TIA, symptoms), surgical (retropharyngeal risk), and diagnostic (angle measurement) dimensions.
- Educational contribution: Reinforces why students need this knowledge in clinical scenarios (e.g., the risk of a coiled ICA during tonsillectomy).

Discussion

Recent reforms in anatomy education have emphasized the necessity of adapting instructional strategies to reduced curricular hours while simultaneously aligning undergraduate training with clinically relevant learning objectives and diverse educational approaches [5,10,22,23]. In response to these developments, anatomy curricula have increasingly incorporated interactive educational methods, including team-based learning, peer-assisted instruction, audience response systems, and simulation-supported teaching environments aimed at improving learner engagement and contextual understanding [6,22–24]. Technological advancements have further expanded these approaches through the integration of radiologic imaging, cross-sectional anatomy, web-based educational resources, and three-dimensional simulation technologies into anatomy instruction [8,15,21,24,25].

Although cadaveric dissection remains a fundamental component of anatomical education, it may present limitations in demonstrating patient-specific anatomical variability and clinically relevant structural differences [2,3]. Furthermore, restricted access to cadaveric materials in some educational settings has increased reliance on atlases and textbook-based instruction, which may not always adequately represent complex three-dimensional anatomical relationships within clinical contexts [26–28]. Previous investigations have shown that 3D anatomical and vascular models can improve perceived spatial orientation, visualization, and learner engagement during anatomy training [17,19,21,28,29]. Similar educational benefits have been reported in simulation-based spinal anatomy training, fracture simulations, vascular reconstruction models, and oncologic 3D educational platforms [12,17,19,21,22,24,26,28-30]. Collectively, these findings support the growing role of simulation-assisted educational tools as complementary com- ponents of contemporary anatomy curricula.

The present study demonstrated that integrating CT-A imaging, patient-specific 3D vascular simulators, and scenario-based anatomy sessions was associated with enhanced student perceptions regarding anatomical visualization and contextual understanding of ICA variations. Participants in the simulator-supported group reported higher levels of perceived educational engagement and learning support across multiple questionnaire domains (Tables 1–4). The findings suggest that clinically oriented scenario discussions combined with realistic vascular models may facilitate the contextualization of anatomical knowledge within undergraduate anatomy education. However, these outcomes should primarily be interpreted as perception-based educational findings rather than objective demonstrations of clinical competency acquisition.

Importantly, the current study did not directly assess objective performance domains such as diagnostic accuracy, clinical reasoning ability, procedural planning, or long-term competency development using validated performance-based assessment instruments. Therefore, statements regarding improvements in "clinical reasoning", "diagnostic preparedness", or "surgical planning" should be interpreted cautiously and within the limitations of self-reported educational evaluation. The questionnaire was specifically designed to assess perceived educational experience, anatomical comprehension, learner confidence, and clinically contextualized understanding rather than measurable professional performance outcomes.

The educational benefits observed in this study should be interpreted as perception-based outcomes derived from students' self-reported experiences rather than objective measures of clinical competence, diagnostic accuracy, or procedural performance. Future studies employing validated performance assessments and longitudinal follow-up are needed to confirm whether these perceived benefits translate into measurable educational gains.

The educational contribution of the simulator-supported intervention appeared particularly related to its ability to represent vascular tortuosity, kinking, coiling patterns, and patient-specific anatomical variation in an interactive and visually accessible manner (Tables 1,2; Figs. 6,7). Students reported that the physical manipulation of the models improved their understanding of anatomical relationships and clinically relevant vascular configurations. Similar observations have been described in previous anatomy education studies evaluating tactile and multisensory simulation environments [17,19,21,28,29]. The opportunity to rotate, examine, and physically interact with vascular structures from multiple perspectives may support spatial comprehension and anatomically contextualized learning [31-34]. Nevertheless, because the present outcomes relied predominantly on questionnaire-based self-assessment, the educational effects observed should not be interpreted as definitive evidence of improved objective clinical performance.

The findings of the present study may also be considered within the broader concept of anatomical diversity. Contemporary anatomical research increasingly recognizes that variability is a fundamental characteristic of human morphology rather than an uncommon departure from a fixed anatomical template. Although educational resources frequently emphasize the most commonly encountered anatomical configurations, clinical practice routinely exposes healthcare professionals to a wide spectrum of structural differences. In this regard, ICA tortuosity, kinking, and coiling patterns should be viewed as components of naturally occurring vascular diversity that may influence anatomical relationships, diagnostic interpretation, and procedural planning [35].

Awareness of such variability is particularly important in vascular anatomy, where alterations in vessel course, branching architecture, carotid bifurcation level, and positional relationships among carotid vessels may affect both surgical orientation and radiological assessment. Previous anatomical investigations have demonstrated that carotid vascular variations may substantially influence operative exposure and increase the complexity of diagnostic and interventional procedures when not recognized beforehand [36]. Educational strategies that expose students to realistic examples of patient-specific vascular configurations may therefore contribute to a more comprehensive understanding of human anatomy beyond idealized textbook representations. By facilitating direct interaction with anatomically diverse models, simulation-supported learning environments may help learners appreciate the clinical relevance of anatomical variation and develop a more realistic perspective on the complexity of human morphology. Accordingly, anatomy education should aim not only to teach standard anatomical patterns but also to foster recognition of the diversity that characterizes human biological structures, a principle emphasized by contemporary evidence-based anatomy approaches [37].

Another important aspect of the intervention was its interdisciplinary educational structure. Students interacted with anatomists, cardiologists, radiologists, and cardiovascular surgeons throughout the scenario-based sessions,

allowing exposure to multiple clinical perspectives. This multidisciplinary framework may have enriched students' perceived understanding of the relationship between anatomical variation and clinical application. However, the independent contribution of interdisciplinary interaction could not be isolated from the educational effects of the 3D simulator models themselves.

Limitations

Several methodological limitations should be acknowledged. First, the study was conducted within a single institution and included a relatively limited cohort of first-year medical students, potentially restricting external validity and broader generalizability. Second, because participant allocation was non-randomized according to the predefined educational framework of the course, selection-related bias cannot be completely excluded. Third, the study primarily evaluated students' perceptions and self-reported educational experiences through questionnaires and focus-group evaluations, introducing the possibility of response-related bias, including Hawthorne and social desirability effects. In addition, long-term knowledge retention, competency progression, and objectively measurable clinical outcomes were not assessed. Therefore, conclusions regarding clinical competency development should remain preliminary and interpreted with methodological caution. Therefore, conclusions regarding clinical reasoning, diagnostic readiness, or surgical competency should be interpreted as perception-based educational observations rather than objectively validated performance outcomes. Furthermore, the study did not include clinicians, surgical trainees, practicing surgeons, or patient-related outcome measures. Therefore, the potential implications of the educational approach for surgical planning, endovascular decision-making, or clinical performance remain speculative and require validation in future clinical and trainee-based investigations. The absence of validated long-term educational assessment tools and multicenter confirmation may further limit the generalizability of the findings.

Despite these limitations, the present study suggests that patient-specific 3D vascular simulators integrated with scenario-based anatomy instruction may provide meaningful complementary educational support within undergraduate anatomy curricula. Rather than replacing conventional anatomy teaching, these technologies may function as supportive educational tools that facilitate visualization of anatomical variations, learner engagement, and clinically contextualized understanding. Furthermore, although patient-specific simulator production requires considerable time and material resources, the reusable structure of these models may support broader institutional applicability over time. Future multicenter investigations incorporating randomized educational designs, validated objective performance assessments, and longitudinal follow-up analyses are warranted to determine whether the perceived educational benefits observed in this study translate into measurable long-term academic and clinical outcomes.

Conclusions

Patient-specific life-size models of cervical ICA anatomical variations appeared to support students' visualization and contextual understanding of complex vascular anatomy. The integration of scenario-based learning with 3D anatomical simulation was associated with positive student perceptions regarding anatomical comprehension, learner engagement, and interdisciplinary educational interaction.

However, these findings were primarily based on questionnaire-derived self-reported perceptions and should be interpreted as educational outcomes rather than objective evidence of clinical competency or performance improvement. Despite these limitations, patient-specific 3D vascular simulators may serve as valuable complementary tools in anatomy education by facilitating anatomical visualization and contextual learning. Further multicenter studies incorporating validated objective assessment methods are warranted.

Abbreviations

The following abbreviations are used in this manuscript:

3D	Three Dimensions
ICA	Internal Carotid Artery
CT-A	Computed Tomography Angiography
DICOM	Digital Imaging and Communications in Medicine
STL	Stereolithography

Acknowledgments
We are thankful to Figen Govsa for her generous mentorship and guidance and to Tahir Yagdi for his clinical support throughout the study process. We also acknowledge the institutional bodies that facilitated ethical approval and data access. The authors take full responsibility for the content of this work.

Authors' contributions
O.B.D., F.G., T.Y., I.D., M.A.O., G.G.: Conceptualization, formal analysis, methodology, writing—review and editing, writing—original draft. O.B.D., I.D., G.G.: Data curation, manuscript figures editing. F.G., T.Y., M.A.O.: Supervision, project administration, validation, visualization. All authors have read and agreed to the published version of the manuscript.

Funding
This study received no external funding.

Data Availability
The datasets generated and/or analyzed during the current study are not publicly available due to ethical and institutional restrictions and participant confidentiality requirements but are available from the corresponding author on reasonable request and with permission from the Izmir Bakircay University Non-Interventional Clinical Research Ethics Committee.

Declarations

Ethical Approval
Ethical approval was obtained from the Izmir Bakircay University Non-Interventional Clinical Research Ethics Committee (Decision No: 1863; Research No: 1843 on 20 November 2024). All procedures undertaken were in strict accordance with the ethical principles outlined in the 1964 Declaration of Helsinki and its subsequent amendments. As this study is not a clinical trial, clinical trial regis-tration is not applicable.

Informed Consent Statement
Informed consent was obtained from all students prior to participation.
Consent for Publication
Written informed consent for publication of the image included in this manuscript was obtained from the individual concerned.

Conflicts of Interest
All the authors certify that they have no potential conflicts of interest with any entity mentioned in this manuscript and that they received no specific financial support for this work.

References

1. Barry DS, Dent JM, Hankin M, Moyer D, Shah NL, Tuskey A, et al. The clinical anatomy and imaging laboratory: vertical integration in the preclerkship curriculum. MedEdPORTAL J. 2019;15:10824.
2. Choi-Lundberg DL, Low TF, Patman P, Turner P, Sinha SN. Medical student preferences for self-directed study resources in gross anatomy. Anat Sci Educ. 2015;9:150–60.
3. McMenamin PG, McLachlan J, Wilson A. Do we really need cadavers anymore to learn anatomy in undergraduate medicine? Med Teach. 2018;40:1020–9.
4. Bruno PA, Love Green JK, Illerbrun SL, Holness DA, Illerbrun SJ, Haus KA, et al. Students helping students: Evaluating a pilot program of peer teaching for an undergraduate course in human anatomy. Anat Sci Educ. 2016;9:132–42.
5. Doomernik DE, van Goor H, Kooloos JGM, Ten Broek RP. Longitudinal retention of anatomical knowledge in second-year medical students. Anat Sci Educ. 2017;10:242–8.
6. Berjano P, Villafañe JH, Vanacker G, Cecchinato R, Ismael M, Gunzburg R, et al. The effect of case-based discussion of topics with experts on learners' opinions: implications for spinal education and training. Eur Spine J. 2017;26(10):2660–5.
7. Zhang J, Liu X, Huang Y, Kong L, Su M, Hu Z. 3D reconstructed models based on real cervical cancer cases for undergraduate gynecological oncology education: a pre- and post-test study. 3D Print Med. 2025;11:7.
8. Lufler RS, Davis ML, Afifi LM, Willson RF, Croft PE. Bringing anatomy to life: evaluating a novel ultrasound curriculum in the anatomy laboratory. Anat Sci Educ. 2022;15:609–19.
9. Cheung RCC, Yang J, Fang C, Leung MF, Bridges SM, Tipoe GL. Show them what they can't see! An evaluation of the use of customized 3D printed models in head and neck anatomy. Anat Sci Educ. 2024;17:379–95.
10. Fasel JHD , Aguiar D, Kiss-Bodolay D, Montet X, Kalangos A, Stimec BV, et al. Adapting anatomy teaching to surgical trends: A combination of classical dissection, medical imaging, and 3D-printing technologies. Surg Radiol Anat. 2016;38:361–7.
11. Henssen D, Karstens J, De Jong G, Duits JD, Boer L, Brink M, et al. Implementing 3D printing and extended reality in anatomy education: Organization, evolution, and assessment of an innovative teaching program in an undergraduate medical school in the Netherlands. Ann Anat. 2025;257:152354.
12. Karakas AB, Govsa F, Ozer MA, Eraslan C. 3D brain imaging in vascular segmentation of cerebral venous sinuses. J Digit Imaging. 2019;32:314–21.
13. Lim KHA, Loo ZY, Goldie SJ, Adams JW, McMenamin PG. Use of 3D printed models in medical education: a randomized control trial comparing 3D prints versus cadaveric materials for learning external cardiac anatomy. Anat Sci Educ. 2016;9:213–21.
14. Ramos-Bossini AJL, Cornejo DL, Guerrero PR, Santiago FR. The educational impact of radiology in anatomy teaching: A field study using cross-sectional imaging and 3D printing for the study of the spine. Acad Radiol. 2024;31:329–37.
15. Ergun E, Simsek B, Kosar PN, Yilmaz BK, Turgut AT. Anatomical variations in branching pattern of arcus aorta: 64-slice CTA appearance. Surg Radiol Anat. 2013;35:503–9.
16. Karacan A, Turkvatan A, Karacan K. Anatomical variations of aortic arch branching: evaluation with computed tomographic angiography. Cardiol Young. 2013;24:485–93.

17. Govsa F, Ozer MA, Sirinturk S, Eraslan C, Alagöz AK. Creating vascular models by postprocessing computed tomography angiography images: a guide for anatomical education. Surg Radiol Anat. 2017;39:905–10.
18. Hadeed K, Acar P, Dulac Y, Cuttone F, Alacoque X, Karsenty C. Cardiac 3D printing for better understanding of congenital heart disease. Arch Cardiovasc Dis. 2018;111:1–4.
19. Yammine K, Violato C. The effectiveness of physical models in teaching anatomy: a meta-analysis of comparative studies. Adv Health Sci Educ Theory Pract. 2016;21:883–95.
20. Böckers A, Mayer C, Böckers TM. Does learning in clinical context in anatomical sciences improve examination results, learning motivation, or learning orientation? Anat Sci Educ. 2014;7:3–11.
21. Govsa F, Yagdi T, Ozer MA, Eraslan C, Alagoz AK. Building 3D anatomical model of coiling of the internal carotid artery derived from CT angiographic data. Eur Arch Otorhinolaryngol. 2017;274:1097–102.
22. Agarwal A, Souza AD, Jyostna B, Shetty A, Bhat N. Introducing 3D printed models of fractures in osteology learning improves clinical reasoning skills among first-year medical students: a pilot study. BMC Med Educ. 2025;25:190.
23. Al-Ani SA, Chandla D, Delieu J, Yu ST, Fratini A, Gkountiou R, et al. Use of 3D foot and ankle puzzle enhances student understanding of the skeletal anatomy in the early years of medical school. Surg Radiol Anat. 2024;46:1429–38.
24. Alaimo L, Marchese A, Vignola D, Roman J, Conci S, De Bellis MD, et al. The role of three dimensional modeling to improve comprehension of liver anatomy and tumor characteristics for medical students and surgical residents. J Surg Educ. 2024;81:597–606.
25. Yang C, Yang X, Yang H, Fan Y. Flipped classroom combined with human anatomy web-based learning system shows promising effects in anatomy education. Medicine (Baltimore). 2020;99:e23096.
26. Bati AH, Guler E, Ozer MA, Govsa F, Erozkan K, Vatansever S, et al. Surgical planning with patient-specific three-dimensional printed pancreaticobiliary disease models - Cross-sectional study. Int J Surg. 2020;80:175–83.
27. Pujol S, Baldwin M, Nassiri J, Kikinis R, Shaffer K. Using 3D modeling techniques to enhance teaching of difficult anatomical concepts. Acad Radiol. 2016;23:507–16.
28. Triepels CPR, Smeets CFA, Notten KJB, Kruitwagen RFPM, Futterer JJ, Vergeldt TFM, et al. Does three-dimensional anatomy improve student understanding? Clin Anat. 2020;33:25–33.
29. Garas M, Vaccarezza M, Newland G, McVay-Doornbusch K, Hasani J. 3D printed specimens as a valuable tool in anatomy education: A pilot study. Ann Anat. 2018;219:57–64.
30. Guler E, Ozer MA, Bati AH, Govsa F, Erozkan K, Vatansever S, et al. Patient-centered oncosurgical planning with cancer models in subspecialty education. Surg Oncol. 2021;37:101537.
31. Cui D, Lynch JC, Smith AD, Wilson TD, Lehman MN. Stereoscopic vascular models of the head and neck: A computed tomography angiography visualization. Anat Sci Educ. 2016;9:179–85.

32. McBride JM, Drake RL. Longitudinal cohort study on medical student retention of anatomical knowledge in an integrated problem-based learning curriculum. Med Teach. 2016;38:1209–13.
33. Wang C, Zhang L, Qin T, Xi Z, Sun L, Wu H, Li D. 3D printing in adult cardiovascular surgery and interventions: a systematic review. J Thorac Dis. 2020;12:3227–3237
34. Ogden KM, Aslan C, Ordway N, Diallo D, Tillapaugh-Fay G, Soman P. Factors affecting dimensional accuracy of 3-D printed anatomical structures derived from CT Data. J Digit Imaging. 2015;28:654–63.
35. Żytkowski A, Tubbs RS, Iwanaga J, Clarke E, Polguj M, Wysiadecki G. Anatomical normality and variability: historical perspective and methodological considerations. Transl Res Anat. 2021;23:100–5.
36. Coffman-Rea K, Arwood S, Samonds KE, Fuchs DJ, Negrin JP. Laterally positioned external carotid arteries: two cadaveric case reports. Transl Res Anat. 2025;38:100381.
37. Wysiadecki G, Varga I, Klejbor I, Balawender K, Ghosh SK, Clarke E, ... Żytkowski A. Reporting anatomical variations: should unified standards and protocol (checklist) for anatomical studies and case reports be established?. Translational Research in Anatomy. 2024;35,100284.
